# Exogenous Application of Glycine Betaine to *Passiflora edulis* Sims f. *flavicarpa* to Mitigate Drought Stress on Two Propagation Methods

**DOI:** 10.3390/ijms26178734

**Published:** 2025-09-08

**Authors:** Leonardo de Almeida Oliveira, Nga Thi Thu Nguyen, Nasratullah Habibi, Maryam Dabirimirhosseinloo, Naoki Terada, Atsushi Sanada, Kaihei Koshio

**Affiliations:** 1 Laboratory of Tropical Horticultural Science, Department of International Agricultural Development, Faculty of International Agricultural and Food Studies, Setagaya Campus, Tokyo University of Agriculture, 1-1-1 Sakuragaoka, Setagaya, Tokyo 156-0054, Japan; leonardoaaloliveira@gmail.com (L.d.A.O.); 13423004@nodai.ac.jp (N.T.T.N.); nasratullah.habibi14@gmail.com (N.H.); 13423403@nodai.ac.jp (M.D.); nt204361@nodai.ac.jp (N.T.); a3sanada@nodai.ac.jp (A.S.); 2 Faculty of Food Science and Technology, Vietnam National University of Agriculture, Trau Quy, Gia Lam, Hanoi 12406, Vietnam; 3 Faculty of Agriculture, Balkh University, Balkh 1701, Afghanistan

**Keywords:** abiotic stress, fruit production, tropical horticulture, osmoprotectant

## Abstract

Glycine betaine (GB) is a compatible solute that enhances plant tolerance to abiotic stresses, yet its role in fruit crops remains insufficiently explored. This study assessed whether GB improves drought tolerance in *Passiflora edulis* Sims f. *flavicarpa*, a crop sensitive to irregular rainfall. A 3 × 2 × 2 factorial design was employed, combining three drought levels (control, mild, and severe), two propagation methods (seedlings and cuttings), and two GB treatments (0 and 100 mM), with 60 plants and five replicates. Plants were grown under controlled conditions, and irrigation was adjusted to maintain target field capacities. Chlorophyll content was monitored daily, and agronomic and physiological traits were measured after 45 days. GB application influenced leaf water dynamics and stress responses. Cuttings generally showed clearer improvements in drought tolerance when treated with GB, while seedlings exhibited more variable outcomes. These differences appear linked to the propagation method and developmental stage as cuttings were at a more advanced phase, whereas seedlings remained vegetative. Overall, the results demonstrate that exogenous GB can enhance drought tolerance in passion fruit, although its effectiveness is propagation-dependent and context-specific, highlighting the need to tailor its application to cultivation practices.

## 1. Introduction

Yellow Sour Passion Fruit (*Passiflora edulis* Sim f. *flavicarpa*), a member of the Passifloraceae family, is a semi-woody climbing vine native to the Atlantic Forest of Brazil. It has successfully spread throughout South and Central America—including Peru, Ecuador, Venezuela, Jamaica, and Guatemala—and is cultivated worldwide in tropical and subtropical regions [1,2]. Its wide adaptation stems from its physiological flexibility and agricultural value, making it the most produced and consumed passion fruit species globally. This predominance is attributed to its higher yield, concentrated acidic pulp suitable for industrial processing, and better acceptance in markets, especially in Brazil [2].

Brazil is the largest producer and consumer of passion fruit worldwide, with approximately 72.7% of production centered in the northeast region. This area is characterized by semi-arid climatic conditions, including low and erratic precipitation and high temperatures [3]. Despite irrigation practices, passion fruit crops in this region experience significant abiotic stresses such as heat and drought, which adversely affect fruit development, pulp quality, and overall productivity compared to plants grown in the more temperate southern and southeastern regions [4,5]. Notably, while mature passion fruit plants display a degree of drought tolerance, critical phenological stages—especially flowering and fruiting—are highly sensitive to water deficits, resulting in flower and fruit abortion, reduced fruit size, and diminished yields [4,5]. This vulnerability emphasizes the importance of developing agronomic strategies to enhance plant resilience and maintain fruit quality under environmental stress.

Plant response to abiotic stress involves complex morphological, physiological, molecular, and biochemical adaptations [6]. Morphologically, plants may alter leaf structure or root architecture to reduce water loss or improve water uptake. Physiological adjustments include changes in relative water content, stomatal conductance, osmotic potential, and water-use efficiency. At the molecular and biochemical levels, plants accumulate compatible solutes such as proline, glycine betaine, and sugars, and activate antioxidant enzymes to detoxify reactive oxygen species (ROS) generated by stress [7]. These responses are vital to maintaining cellular homeostasis and protecting photosynthetic machinery under drought and heat stress [8,9].

Among various plant growth regulators (PGRs) and osmoprotectants investigated for stress mitigation—including abscisic acid, salicylic acid, jasmonic acid, ethylene, proline, and ascorbic acid—glycine betaine (GB) stands out as a potent small molecule that stabilizes membranes, proteins, and photosynthetic complexes [7,10]. GB is a quaternary ammonium compound naturally accumulated in some plants and can be applied exogenously to enhance stress tolerance [8]. It plays multiple roles, including osmotic adjustment, the protection of photosystem II, activation of heat-shock proteins, and enhancement of antioxidant defenses, thereby reducing oxidative damage caused by drought, heat, and cold stresses [10].

Despite the promising properties of GB demonstrated in short-cycle crops such as wheat, maize, and tomato, its application in perennial fruit crops like passion fruit remains underexplored [10]. Reference [11] provided some evidence of GB mitigating cold stress in passion fruit, showing improved survival rates under frost conditions at different altitudes. Their findings suggest that GB’s protective effects are transient and depend on application timing relative to stress periods. Research on GB’s effectiveness against drought stress in passion fruit is even more limited, although studies on other fruit crops like cucumber [12], summer squash [13], and strawberry [14] have reported an enhanced growth, yield, and stress tolerance following GB treatment.

Abiotic stresses such as drought, salinity, and cold impose complex constraints on plant growth, triggering adjustments at morphological, physiological, and molecular levels. In addition to osmolytes like glycine betaine that stabilize cellular homeostasis, transcriptional regulation has emerged as a critical determinant of stress tolerance. A wide range of transcription factors (TFs) and functional genes orchestrate downstream pathways that control osmotic adjustment, antioxidant activity, and hormonal signaling, thereby shaping plant responses to adverse environments.

Members of the ICE, MYB, and WRKY TF families have been particularly well documented in this regard. In *Malus baccata*, MbICE1, MbMYBC1, and MbWRKY50 were shown to regulate stress-responsive gene expressions and enhance tolerance to drought and cold stresses [15,16,17]. Similarly, in *Fragaria vesca*, the MYB TFs, FvMYB114 and FvMYB44, contribute to protective mechanisms under abiotic stress conditions [18,19]. In the grapevine (*Vitis* species), VhMYB2 and VhWRKY44 have also been implicated in regulating molecular pathways associated with water deficits, salinity, and low-temperature tolerance [20,21]. Collectively, these studies emphasize that abiotic stress tolerance is not solely dependent on osmoprotectants but also relies heavily on TF-mediated transcriptional reprogramming. This broader context underscores the need to evaluate both metabolic and molecular contributions to stress adaptation when assessing the role of exogenous GB in passion fruit.

Furthermore, passion fruit plants propagated through seedlings and cuttings exhibit notable morphological and physiological differences that could influence their response to stress and GB treatment [22,23]. Seedlings, arising from sexual reproduction, have greater genetic variability, which can confer advantages in adapting to environmental stresses but may result in unpredictable productivity. Conversely, cuttings produce genetically uniform plants with consistent traits but potentially reduce stress resilience due to limited genetic diversity [24]. Limited studies have compared how these propagation methods interact with abiotic stress and PGR applications, highlighting a crucial knowledge gap for passion fruit agronomy.

In conclusion, passion fruit production is economically significant but constrained by abiotic stresses, particularly drought and heat in Brazil’s semi-arid regions. Glycine betaine presents a promising means to improve stress tolerance, yet its effects on passion fruit—especially considering the propagation method and phenological stage—are not fully understood. This study aims to evaluate the impact of exogenous GB applications on passion fruit plants propagated by seedlings and cuttings during vegetative and reproductive phases, contributing to optimized management practices to enhance yield and quality under stress conditions.

## 2. Results

### 2.1. Effect of Glycine Betaine on Water Relations and Leaf Chlorophyll Content

In the Results, Discussion, and figures, several abbreviations are used: “S” represents seedlings, “C” represents cuttings, “D0” refers to control (no drought), “D1” indicates mild drought, and “D2” denotes severe drought. “GB0” corresponds to no glycine betaine application, whereas “GB1” signifies glycine betaine application.

**Figure 1:**
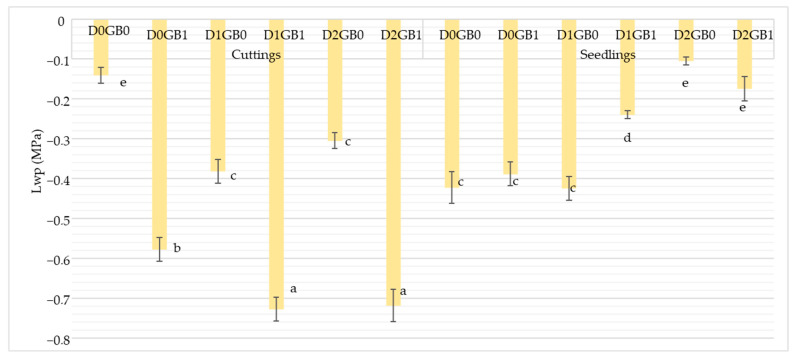
Effect of GB (glycine betaine) application on leaf water potential of passion fruit cuttings and seedlings. “S” represents seedlings, “C” represents cuttings, “D0” refers to control (no drought), “D1” indicates mild drought, and “D2” denotes severe drought. “GB0” corresponds to no glycine betaine application, whereas “GB1” signifies glycine betaine application. Different letters mean different significant results with Tukey at 5%.

Statistically significant differences were observed in leaf water potential (Lwp) measurements using the potentiometer (Figure 1). In seedlings, Lwp values were generally higher (less negative) than in cuttings. In cuttings, control plants maintained the highest water potential, mild drought (D1GB0) produced the lowest values, and severe drought (D2GB0) showed slightly higher values than mild drought. With GB treatment, cuttings often exhibited intermediate values (D1GB1, D2GB1) without a consistent trend toward improvement. In seedlings, a different pattern was observed: control plants (D0GB0) showed the lowest Lwp, while both mild (D1GB0) and severe drought (D2GB0) maintained comparatively higher values. GB application in seedlings produced variable responses without consistent improvement. Overall, the effect of GB on Lwp was propagation-dependent, with a clearer separation of stress treatments in cuttings than in seedlings.

**Figure 2:**
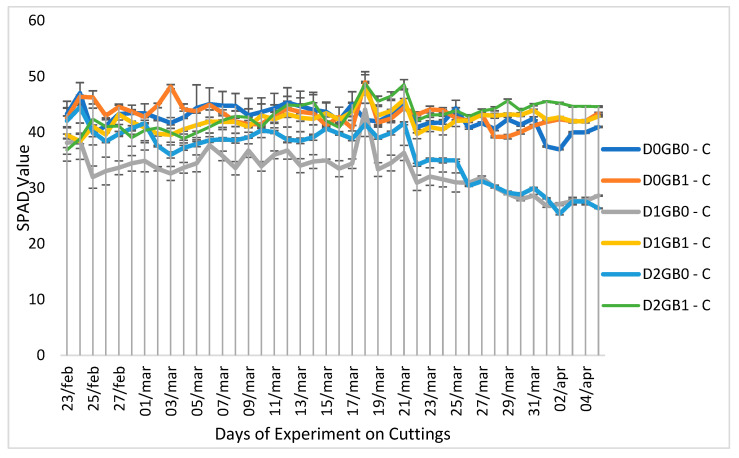
Effect of GB application on SPAD values of cuttings of passion fruit. “S” represents seedlings, “C” represents cuttings, “D0” refers to control (no drought), “D1” indicates mild drought, and “D2” denotes severe drought. “GB0” corresponds to no glycine betaine application, whereas “GB1” signifies glycine betaine application.

**Figure 3:**
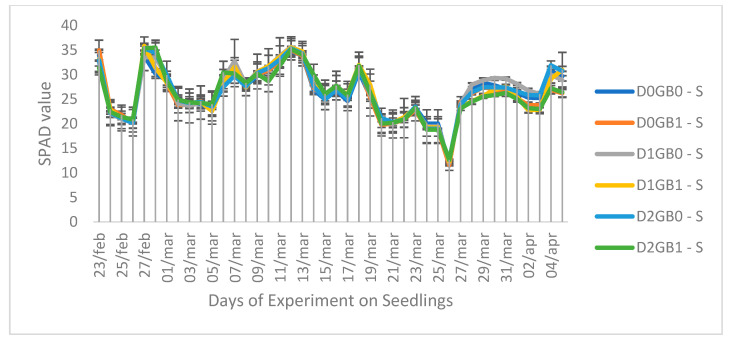
Effect of GB application on SPAD values of seedlings of passion fruit. “S” represents seedlings, “C” represents cuttings, “D0” refers to control (no drought), “D1” indicates mild drought, and “D2” denotes severe drought. “GB0” corresponds to no glycine betaine application, whereas “GB1” signifies glycine betaine application.

The soil–plant analysis component (SPAD) values, reflecting chlorophyll content in leaves, showed significant differences between cuttings (Figure 2) and seedlings (Figure 3). Seedlings generally had lower SPAD values than cuttings, which may be attributed to their developmental stage, as seedlings were still in the vegetative phase while cuttings were entering the reproductive stage where leaf structural development is greater. In cuttings, severe drought without GB (D2GB0-C) produced the lowest SPAD values, whereas GB application under severe drought (D2GB1-C) maintained a higher SPAD, indicating a protective effect of GB. Control and mild drought treatments remained at intermediate levels. In seedlings, SPAD values across treatments were closely clustered, with no consistent separation among drought or GB levels, suggesting that SPAD was not a sensitive indicator in this propagation type.

After 4 months of treating the passion fruits, SPAD was measured again, especially due to the occurrence of summer, which could modify the metabolism of the plants. The results show that after the mentioned period, the differences in SPAD between the treatments became even more significant. All of the treatments that were applied with glycine betaine were significantly superior to the ones that did not use an osmoprotectant, even those on the seedlings that, before, showed stable trends for SPAD values.

**Figure 4:**
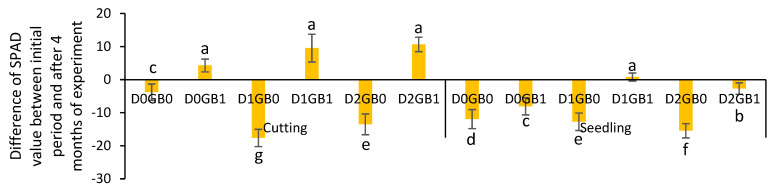
Effect of GB on the difference in SPAD values of seedlings and cuttings between the initial value on the first day, and the reading after 4 months of treatment. Different letters mean different significant results with Tukey at 5%. S” represents seedlings, “C” represents cuttings, “D0” refers to control (no drought), “D1” indicates mild drought, and “D2” denotes severe drought. “GB0” corresponds to no glycine betaine application, whereas “GB1” signifies glycine betaine application. Different letters mean different significant results with Tukey at 5%.

After discovering the SPAD values for 4 months of treatment, the difference between that value and the first value found at the start of the experiment was calculated. The results, shown in Figure 4, note that all of the treatments with GB application showed a better response than their counterpart and provided a clearer vision of the pathway that the treatments traversed during these months.

### 2.2. Effect of Glycine Betaine on Stomatal Behavior, Cuticle Thickness, and Transpiration

**Figure 5:**
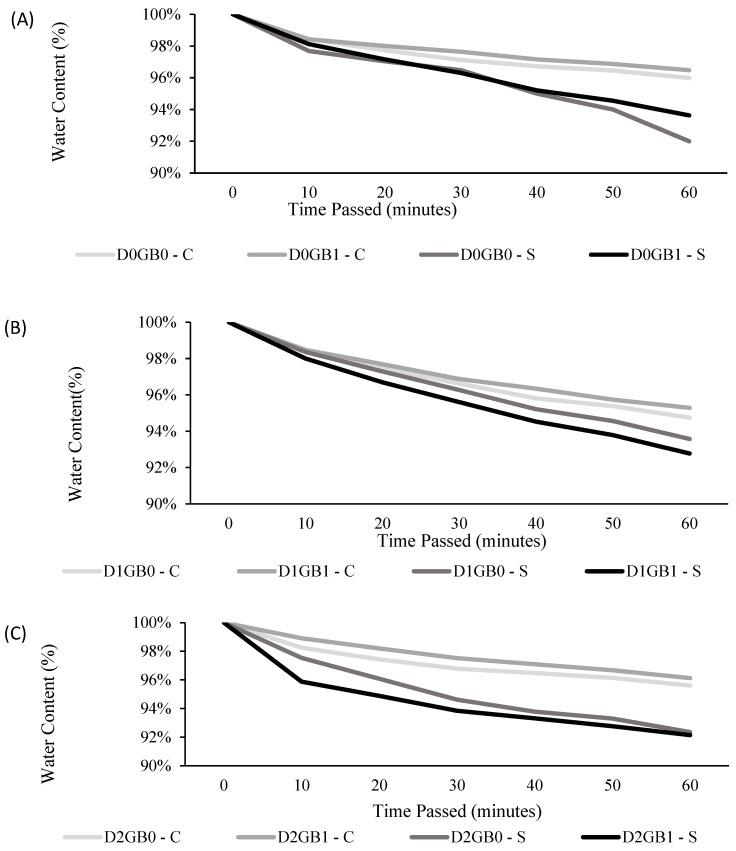
(**A**) Effect of GB application on the hourly transpiration rate of control drought of the cuttings and seedlings of passion fruit (D0); (**B**) effect of GB application on the hourly transpiration rate of mild drought of the cuttings and seedlings of passion fruit (D1); and (**C**) effect of GB application on the hourly transpiration rate of severe drought of the cuttings and seedlings of passion fruit (D2). “S” represents seedlings, “C” represents cuttings, “D0” refers to control (no drought), “D1” indicates mild drought, and “D2” denotes severe drought. “GB0” corresponds to no glycine betaine application, whereas “GB1” signifies glycine betaine application.

To observe the water dynamics of passion fruit leaves under different treatments after detachment, hourly transpiration rates were measured for the control (Figure 5A), mild drought (Figure 5B), and severe drought (Figure 5C). Under control conditions, all groups displayed similar stomatal closure times, suggesting that GB did not substantially alter leaf water dynamics in the absence of stress. However, seedlings exhibited slightly higher cuticular transpiration compared to cuttings, which may be attributed to anatomical differences such as a thinner cuticle or differences in stomatal behavior. Within seedlings, the application of GB produced only a minor difference compared to untreated controls, consistent with the idea that under non-stressful conditions, GB does not strongly influence transpiration dynamics. This difference contrasts with cuttings, which appeared to respond more quickly to stress, possibly due to the faster activation of stress signaling pathways or thicker cuticular layers that reduce cuticular transpiration losses.

In the mild drought treatment, stomatal closure occurred at comparable times across treatments, and cuticular transpiration rates were also similar. This suggests that the inherent drought tolerance mechanisms of passion fruit—particularly under moderate stress—were sufficient to buffer differences between GB-treated and untreated plants. In other words, GB did not cause a measurable divergence in water loss under mild drought, likely because oxidative stress levels were still within the range that plants could regulate through basal defense mechanisms.

In the severe drought treatment, stomatal closure became much more pronounced and differences between propagation methods became clearer. Cuttings maintained reduced transpiration after GB application, supporting the idea that GB enhanced water conservation in this propagation type under high stress. By contrast, seedlings—particularly those treated with GB—showed a delayed stomatal closure and greater water loss over the 60 min test. While this might initially appear contradictory, it may reflect differences in physiological plasticity between propagation methods: seedlings, derived from sexual reproduction, exhibit greater genetic variability and may respond more heterogeneously to GB compared to clonal cuttings. Additionally, seedlings may allocate resources differently under stress, prioritizing growth or other metabolic functions at the expense of rapid stomatal regulation. As a result, GB application did not uniformly enhance seedling drought responses in terms of water content, though it may still influence other physiological parameters.

### 2.3. Adverse Effect of Glycine Betaine

**Figure 6:**
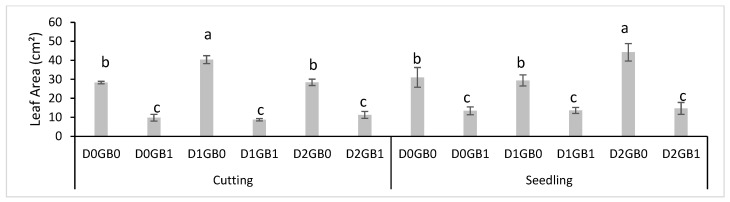
Effect of GB application on leaf area values of cuttings and seedlings of passion fruit. Different letters mean different significant results with a Tukey at 5%. “D0” refers to control (no drought), “D1” indicates mild drought, and “D2” denotes severe drought. “GB0” corresponds to no glycine betaine application, whereas “GB1” signifies glycine betaine application. Different letters mean different significant results with Tukey at 5%.

The role of the leaf area in assessing plant stress is critical, as drought typically reduces the leaf area to minimize water loss through transpiration, which, in turn, affects photosynthesis [25]. In this study, no immediate differences in the leaf area were observed during the experiment. However, after six months of frequent GB application, a noticeable decrease in the leaf area was observed in the passion fruit plants, as shown in Figure 6. This suggests that GB application might damage overall plant growth when applied for a long period of time at the concentrations of 100 mM and, possibly, beyond.

### 2.4. Metabolic Responses to Stress

After making the metabolite assessment on the leaf tissues of passion fruit under the experiments and analyzing it with statistical tools, it was clear that some metabolites had more impact on their concentration in comparison to others. From the 53 metabolites analyzed, the 9 that were chosen to be discussed further were leucine, valine, succinic acid, malic acid, threitol, aminobutyl, citric acid, glucose, and proline, with the results being shown in Table 1.

**Figure 7:**
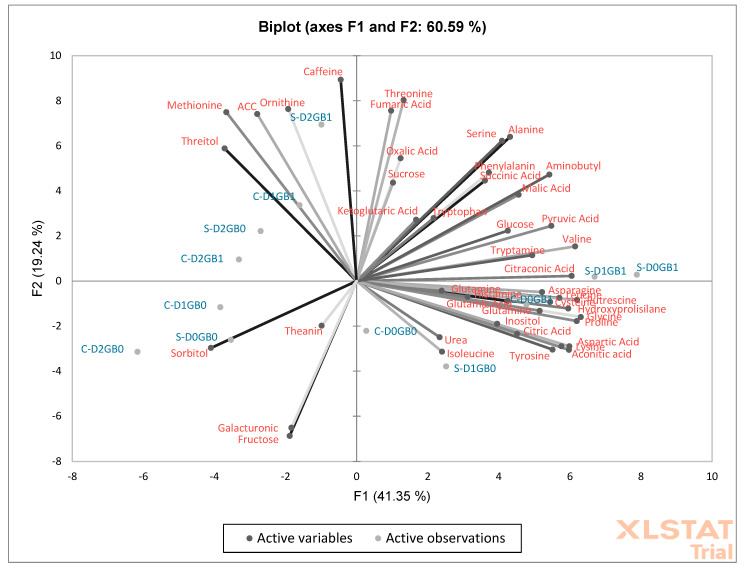
Principal Component Analysis (PCA) of the 50 metabolites in relation to the treatments of the experiment. “S” represents seedlings, “C” represents cuttings, “D0” refers to control (no drought), “D1” indicates mild drought, and “D2” denotes severe drought. “GB0” corresponds to no glycine betaine application, whereas “GB1” signifies glycine betaine application.

The Principal Component Analysis (PCA) biplot (Figure 7) reveals distinct patterns in the metabolic responses of plants under varying conditions of drought severity, glycine betaine application, and plant propagation type. Seedlings generally exhibit more pronounced metabolic adjustments than cuttings, particularly when glycine betaine is applied. Treatments such as S-D1GB1 and S-D2GB1 are positioned in the biplot near metabolites linked to key stress-response pathways, including nitrogen assimilation (e.g., glutamine and asparagine), energy metabolism (e.g., glucose, pyruvic acid), and oxidative stress mitigation (e.g., proline) [26,27,28,29]. These responses highlight the role of glycine betaine in enhancing metabolic adaptation, particularly under conditions of moderate and severe drought.

## 3. Discussion

### 3.1. Role of Glycine Betaine in Water Relations and Leaf Chlorophyll Content

Previous studies on passion fruit have reported variable responses of leaf water potential (Lwp) under stress. For instance, [30] observed no significant differences between drought-stressed and control plants, while [31] found that salinity stress reduced the Lwp, consistent with the physiological overlap between drought and salinity [32,33]. In other crops such as wheat, GB application has been shown to increase Lwp under drought [34]. In our study, however, the patterns did not uniformly follow the expected trend of a progressively lower Lwp with increasing drought severity. In cuttings, mild drought produced the lowest values, while severe drought was not consistently lower, suggesting the influence of osmotic adjustment or other compensatory mechanisms. In seedlings, control plants displayed unusually low values compared with stressed treatments, and, overall, seedlings maintained higher (less negative) water potentials than cuttings. This may reflect differences in the developmental stage, as seedlings were still in the vegetative phase with a smaller canopy size, while cuttings had entered the reproductive stage, where higher transpirational demands can lower water potential. GB application did not consistently increase Lwp, but its influence appeared to be propagation-dependent, with cuttings showing clearer drought responses than seedlings. Altogether, while Lwp alone was not a conclusive indicator of GB-mediated drought tolerance in passion fruit, complementary indicators such as SPAD stability, leaf water content, and transpiration dynamics provide stronger evidence that GB supports drought resilience, particularly in cuttings.

The SPAD response in cuttings (Figure 2) clearly showed that GB buffered chlorophyll loss under severe drought. While severe stress without GB (D2GB0-C) produced the lowest SPAD values, GB-treated cuttings under severe drought (D2GB1-C) maintained higher values, consistent with the protective role of GB in sustaining chlorophyll content and, indirectly, the photosynthetic apparatus. By contrast, in seedlings (Figure 3), SPAD values remained tightly clustered across treatments, with no consistent differences among drought levels or GB application. This suggests that, in seedlings, SPAD is not a sensitive indicator of stress, possibly due to their still-developing roots and protective structures.

Previous studies on GB and SPAD have reported variable results. For example, [35] observed that GB application under drought decreased SPAD values in wild blueberries, whereas [36] found no significant changes. The latter is more consistent with our findings in seedlings, where SPAD remained stable regardless of GB treatment. However, in cuttings, GB application under severe drought maintained the chlorophyll content compared to the non-GB treatment, highlighting a clear propagation-dependent effect. Together, these results suggest that while SPAD is not a direct indicator of the photosynthetic rate in passion fruit seedlings, GB can contribute to maintaining chlorophyll stability in cuttings exposed to severe drought.

### 3.2. Stomatal Behavior, Cuticle Thickness, and Transpiration

Previous studies have shown that glycine betaine (GB) can influence leaf water dynamics under drought stress. For example, [36] reported that GB application in *Andrographis paniculata* increased transpiration (E) both under control and drought conditions, suggesting that GB may help maintain the stomatal opening and improve water-use efficiency during stress. In our study, cuttings treated with GB under severe drought showed a tendency to conserve water through reduced transpiration, whereas seedlings exhibited the opposite trend, with GB-treated seedlings losing water more rapidly. Although this may initially appear contradictory, it highlights that the effect of GB, which may be strongly dependent on the propagation method and genetic variability, since seedlings derived from sexual reproduction are more heterogeneous than clonal cuttings.

Stomatal closure dynamics were inferred indirectly from the temporal decline in leaf fresh mass after detachment (a proxy for transpiration), providing a relative comparison among treatments. Direct gs measurements were not performed in this study. While stomatal conductance (gs) measurements are the standard approach to quantify this process [37,38], our method provided a relative comparison of closure timing between treatments. Future studies incorporating direct gs measurements would allow a more precise quantification of stomatal behavior.

The role of the cuticle is also central to these dynamics. As a barrier to water vapor diffusion, a thicker cuticle reduces cuticular transpiration, thereby lowering water loss when stomata are closed or partially closed. This is consistent with previous studies emphasizing the role of cuticle thickness in limiting non-stomatal water loss [39,40]. In our study, seedlings showed a higher cuticular transpiration than cuttings, which may partly explain their different responses to GB under severe drought.

Overall, GB’s protective role appears to operate indirectly through stabilizing membranes and proteins, maintaining stomatal function, and possibly influencing cuticle properties [7,41]. However, the contrasting responses observed between propagation methods indicate that GB effects on drought tolerance are not uniform, and further research under longer-term and field conditions is needed to fully clarify these mechanisms.

### 3.3. Potential Phytotoxicity of Glycine Betaine

What was visibly found after 6 months of the experiment was that the leaves from the treatments with the glycine betaine application began to grow small and shriveled even in control drought treatments, which begets that this is a reaction to GB itself and not to the water treatments. This reaction may be due to the certain toxicity of GB in the passion fruit plant under the concentration or frequency that they were applied, or even that this amino acid should only be applied for short periods when drought is happening. This proves that more research should be performed to perceive a better concentration and frequency of application of glycine betaine on plants, considering the positive results in agronomic and physiological responses presented in the tables prior.

### 3.4. Amino Acids and Metabolic Responses to Stress

Valine is an amino acid essential for protein synthesis in plants and therefore plays a critical role in plant health. Within plants, valine, along with glutamine, contributes to the formation of proteins that are sensitive to oxidative stress, helping defend plants against both biotic and abiotic stresses and supporting overall development [42]. Another amino acid involved in oxidative stress responses is leucine, which is present in stress defense pathways as part of bZIP (basic leucine zipper) transcription factors. These transcription factors represent one of the largest regulatory groups associated with osmotic stress in many plants, playing important roles in response to abiotic stress [43].

In this study, leucine concentrations (Table 1) showed a stronger response in cuttings subjected to drought, being significantly higher under both mild and severe drought treatments, while the control concentration was nearly 0 µmol/g. In seedlings, a natural rise in leucine concentration was observed during the mild drought period, statistically similar to levels in GB-treated plants. Under severe drought, leucine levels in seedlings became significantly higher compared to GB-treated plants, suggesting that leucine plays a major role in the natural oxidative stress defense of passion fruit.

The amino acids discussed here, particularly the branched-chain amino acids (BCAAs), have gained increasing attention in plant stress science. BCAAs, including valine, isoleucine, and leucine, are known to accumulate under oxidative stress conditions, such as drought and high salinity, often more than other stress-related amino acids like proline. This trend was observed by [44] in several plant species and further supported by [45] and [46], who reported the upregulation of BCAAs under drought stress in *Oryza sativa* and *Sesamum indicum*, respectively. These findings highlight the growing importance of BCAAs in mitigating oxidative stress.

In addition to their role in stress tolerance, BCAAs have been proposed to serve as alternative electron donors in mitochondrial respiration, enhancing energy production under stress [47,48]. Moreover, several studies have shown that glycine betaine (GB), a well-known osmoprotectant, can increase BCAA levels and improve chlorophyll content and photosynthetic performance, thereby enhancing plant resilience under stress [49,50,51].

In the current study, valine stood out as the metabolite with the most significant differences among treatments, exhibiting seven distinct statistical variations across propagation methods and GB applications (Table 1). These results suggest that valine content in passion fruit plants is highly influenced by both the propagation method and GB treatment, positioning valine as a key component in the plant’s defense against oxidative stress.

### 3.5. Organic Acids and Osmotic Defense

Organic acids, such as succinic acid, malic acid, and galacturonic acid, are crucial for plants’ osmotic defense against abiotic stresses like drought. Succinic acid, in particular, is known to accumulate during stress conditions, contributing to the plant’s stress response. References [52,53] noted that succinic acid plays a protective role by participating in the citric acid cycle, helping plants cope with oxidative stress. In the current experiment, succinic acid concentrations were generally higher in treatments involving GB, except in the cuttings under the D1GB1 treatment. Additionally, cuttings, which were propagated differently, had lower baseline succinic acid levels compared to seedlings and were less responsive to GB application.

Malic acid, another important organic compound, also increases significantly under stress, particularly drought. Malic acid participates in photosynthetic metabolism and plays a critical role in energy production within the plant [54]. Research by [55] has shown that malic acid, along with other organic acids like fumaric and citric acid, accumulates in plant leaves during drought stress, likely to support the energy cycle. In this study, all treatments involving GB application resulted in higher malic acid concentrations in passion fruit leaves compared to the control, with seedlings showing particularly higher levels. This suggests that foliar-applied GB can enhance the production of organic acids related to osmotic defense, supporting the plant’s response to stress.

### 3.6. Sugar Alcohols and Non-Protein Amino Acids in Stress Responses

Threitol, a sugar alcohol known for its rapid accumulation during drought stress, was also examined. Reference [56] reported that threitol serves as an important osmoprotectant in various plant species. In the current experiment, seedlings showed a stronger response to GB application, especially under severe drought conditions, with increased threitol concentrations. In contrast, cuttings responded more positively to GB application under mild stress, suggesting that the physiological state of the plant (seedling vs. cutting) can influence the dynamics of this osmoprotectant.

Gamma-aminobutyric acid (GABA), a non-protein amino acid, has also been implicated in plant stress responses. It promotes nitrogen recycling, improves chlorophyll content, protects photosystem II, and helps regulate osmotic pressure [57,58]. In this study, GB application led to a significant increase in aminobutyl (a precursor of GABA) concentrations across all treatments, particularly in cuttings. This suggests that GB application may influence GABA levels in plants, thereby enhancing their ability to cope with oxidative stress. Because both GABA and GB are associated with maintaining chlorophyll content under drought, this link may partly explain the higher SPAD values observed in GB-treated cuttings.

### 3.7. Citric Acid and Soluble Sugars in Plant Stress

Citric acid, a key intermediate in the tricarboxylic acid cycle, also plays a critical role in plant stress responses. It participates in respiration and can help adjust the pH of the cytosol during drought stress, which is crucial for maintaining metabolic activity under conditions that limit gas exchange and respiration [59]. The current study showed that citric acid levels rose considerably in both seedlings and cuttings under severe drought stress. However, under mild drought conditions, treatments with GB application exhibited lower citric acid concentrations, suggesting that citric acid might be more effective in mitigating extreme stress conditions.

Soluble sugars, such as sucrose, fructose, and glucose, are essential for energy production and osmoregulation in plants. Reference [60] found that the concentration of soluble sugars increases during stress, acting as both an energy source and osmolyte to stabilize cellular functions. In this study, glucose concentrations in the leaf tissues of passion fruit were significantly higher in the seedlings following GB application, particularly under mild drought conditions. Conversely, cuttings, which had already undergone some stress during their propagation, showed relatively stable glucose levels, indicating a more balanced response to stress.

Reference [61] found that the application of GB to sweet potatoes under water-deficit conditions led to an increase in sugar levels, particularly glucose. This aligns with the findings of the current study, where GB treatment promoted higher glucose concentrations in seedlings under mild drought stress. Interestingly, the response to GB in cuttings was more similar to the results found in the sweet potato study, where glucose levels were higher in the control group under severe drought.

### 3.8. Proline and Other Osmoprotectants

Proline, another critical amino acid, is well known for its protective role in stress tolerance. It acts as a chaperone for proteins, maintains cell turgor, and enhances enzyme activity during stress [26,28]. In this study, proline levels were found to be low or absent in the leaf tissues of passion fruit under control conditions, with a significant increase following GB application under mild drought. Interestingly, under severe drought, proline levels were higher in plants not treated with GB, suggesting that proline may serve as an early defense mechanism against stress but may be less critical under prolonged or severe stress.

In contrast, treatments without glycine betaine, such as C-D2GB0 and S-D0GB0, were associated with less diverse metabolic responses, relying more heavily on osmoprotectants such as sorbitol, fructose, and galacturonic acid [62,63]. These metabolites clustered toward the negative axes, indicating a limited capacity to modulate complex metabolic pathways in response to stress. Severe drought, in particular, elicited significant metabolic shifts that were amplified when glycine betaine was applied, underscoring its role as a key enhancer of drought tolerance.

The propagation method also played a critical role, with seedlings showing a greater ability to respond dynamically to stress than cuttings. Seedlings treated with glycine betaine were associated with an increased activation of stress-related metabolites, whereas cuttings tended to cluster near less active metabolic regions regardless of drought severity. Mild drought conditions stimulated intermediate metabolic responses, showing the partial activation of energy and stress-response pathways, whereas control drought conditions showed minimal changes in metabolic activity, reflecting a baseline state.

Some of the research limitations include the controlled greenhouse conditions, which may not fully reflect the complexity and variability of field environments where multiple biotic and abiotic factors interact simultaneously. The use of plants at different phenological stages, combined with distinct propagation methods, makes it challenging to clearly attribute observed effects to either developmental stage or propagation technique. Additionally, the short duration of the experiment limits the ability to assess long-term impacts on plant yield and health. It is also important to note that seedlings, derived from sexual reproduction, exhibit significant genetic variability, whereas cuttings are genetic clones, potentially leading to different physiological responses and limiting the generalizability of the results. Future studies with longer durations and field conditions are recommended to deepen the understanding of these variables and optimize management strategies. A limitation of the present study is that reactive oxygen species (ROS) levels and antioxidant enzyme activities were not measured. These parameters could provide additional insight into the physiological mechanisms underlying glycine betaine-mediated drought tolerance in passion fruit. Future studies should include these analyses to further elucidate the biochemical pathways involved.

Future studies should also replicate this experiment under various abiotic stresses such as cold, heat, salinity, and freezing conditions to evaluate the broader effectiveness of glycine betaine (GB) in passion fruit stress tolerance. Testing a range of GB concentrations will be important to identify optimal doses for different plants and to assess any potential toxicity under each stress type. Moreover, extending these experiments from controlled greenhouse environments to field conditions will be essential to validate the practical applicability and performance of GB treatments in real agricultural settings. It would also be important to clearly differentiate between treatments by ensuring that plants from different propagation methods are at the same phenological stage.

## 4. Materials and Methods

### 4.1. Experimental Design

A completely randomized design (factorial 2 × 3 × 2) was used, with two propagation methods: cuttings (removed from passion fruit plants at the reproductive stage)—(C), and seedlings (analyzed at the vegetative stage)—(S); two glycine betaine (GB) treatments: control (without GB application)—(GB0), and GB application at the optimal dose determined in a pre-trial experiment—(GB1); and three drought stress management levels: control (without drought)—(D0), mild drought (50% of the field capacity during the study period)—(D1), and severe drought (no irrigation during the study period)—(D2). There were five replications per treatment, totaling 60 experimental units. Statistical analyses were performed using InfoStat software version 2020d, and mean differences were assessed with Tukey’s test at a 5% significance level.

Prior to the main experiment, two pre-experiments were conducted to determine the optimal concentration and application frequency of glycine betaine (GB) for passion fruit plants under drought-stress conditions. The first pre-experiment involved applying eight different GB concentrations (0, 2, 5, 10, 20, 50, 100, and 200 mM) to 3-month-old passion fruit seedlings subjected to simulated drought, with five replicates per treatment. The second pre-experiment assessed the effect of application frequency on 1-year-old passion fruit plants using the same concentrations, with GB applied weekly, bi-weekly, monthly, or as a single application. For both pre-experiments, 10 leaves per plant were selected to monitor GB uptake and evaluate potential phytotoxicity. Based on these results, the optimal GB concentration (100 mM) and application frequency were selected for use in the main trials to maximize stress mitigation while avoiding toxicity.

### 4.2. Experimental Indexes

The indexes of the experiment were divided into agronomic and physiological categories. The agronomic indexes included leaf fresh weight, leaf dry weight, and leaf area, all measured four months after the end of the initial experiment. The physiological indexes included water potential, SPAD values during the 45 days of the experiment (except the first three days), SPAD values four months after the experiment, the difference between initial SPAD values and those obtained after four months, the hourly transpiration rate, and the concentrations of valine, succinic acid, malic acid, threitol, aminobutyl, citric acid, glucose, and proline. The SPAD value, obtained using a SPAD chlorophyll meter (Konica Minolta, Tokyo, Japan), is a non-destructive indicator of leaf chlorophyll content, which is commonly used to evaluate plant health and nitrogen status. While higher SPAD values may suggest improved maintenance of the photosynthetic apparatus, they should not be interpreted as a direct measurement of the photosynthetic rate.

### 4.3. Experiment Management

The seeds were sown in germination trays and transplanted to vases according to the size and phenological stage of the plants. The plants were grown in pots filled with Akadama Soil (Ibaraki Akadama Soil, Ibaraki, Japan), a volcanic clay substrate commonly used for good drainage and aeration. Fertilization was carried out via foliar spraying with a mix of 8 g of OAT house fertilizer and 6 g of OAT house fertilizer 2, applied three times a week, starting one month after planting both cuttings and seedlings. When the cuttings reached four months and the seedlings reached six months, the height and development of the plants were similar and the experiment began.

The drought stress treatments were conducted by weighing the water applied to reach the desired percentage of field capacity for each treatment. At the beginning of the experiment, treatments were applied and the leaf chlorophyll content (SPAD-502) was measured daily. In the severe drought treatment, rehydration occurred only when wilting of the plant was evident. SPAD measurements were taken again four months after the experiment ended to evaluate the lasting effects of glycine betaine over time. The difference between the initial and final SPAD values was also calculated. During each drought stress period, GB was applied via foliar spraying at the initiation of the drought period and every 10 days thereafter until the experiment ended (45 days) to assess its importance in mitigating oxidative stress under drought.

Four months after the end of the initial experiment, it was noted that leaves from GB0 and GB1 differed, with GB0 plants having unaffected leaves and GB1 plants showing shriveled, smaller leaves. Therefore, a leaf area measurement between the plants was conducted.

### 4.4. Experiment Measurement

Leaf area was measured by the leaf area machine (Hayashi Denko Co. Ltd., Tokyo, Japan, AAM-9A) at the Tropical Crop Science Laboratory at the Tokyo University of Agriculture. The leaves chosen for this experiment were the third ones from up down to represent the newer leaves in the experimental units, with the results being given in cm^2^.

The fourth leaf from each plant in the experiment was cut with a razor in the early morning, stored in a previously humidified plastic bag, and brought to the tropical crop science laboratory. There, the pressure chamber instrument (model 1505D—PMS instrument company, Albany, Oregon, USA) was used on each leaf, putting them upside down and raising the pressure inside the chamber until droplets of water could be seen emerging from the fresh cut stems. The pressure was then taken note of.

For hourly transpiration, the third leaf from each experimental unit was removed with a razor in the early morning in a previously humidified plastic bag to keep the humidity until they were brought to the Tropical Horticultural Science Laboratory. The leaves were separated within each treatment and repetition and weighed for the initial weight and then again every 10 min until 60 min to note the amount of weight lost (due to transpiration, both stomatal and cuticular) within an hour.

The processing for the concentration of metabolites is as follows: fresh leaves were collected from each experiment and were brought to the Tropical Horticultural Science Laboratory of the Tokyo University of Agriculture. They were then put in the vacuum pump machine (Tokyo Rikakikai Co. Ltd. (EYELA), Tokyo, Japan, EYELA FDM-100 for 3 days, where they were ready to be crushed. They were then weighed at 100 mg in a 2 mL tube, and 1 pearl was placed in each tube.

Two hundred and fifty µL of methanol was put into each sample, and then they were put in a shaking machine (Retsch GmbH, Haan, Germany, RETSCH MM400) in Tropical Crop Laboratory for 2 min at the frequency of 27 shakings/second. The tubes were, after that, shaken in the small centrifuge (Nichiryo Co., Ltd., Tokyo, Japan, NICHIRYO C1008-B), and 250 µL of chloroform was added to each sample. The tubes with chloroform were transferred to a thermomixer (Eppendorf AG, Hamburg, Germany, Eppendorf thermomixer F2.0) for 3 min at 37 °C. 1200 rpm.

Fifty µL of Ribitol was implemented, as well as ultra-pure water in the volume of 175 µL, vortexing (Scientific Industries, Inc., Bohemia, NY, USA, VORTEX GENIE 2) the mixture until it mixed. After this procedure, the samples were centrifuged (Tomy Seiko Co., Ltd., Tokyo, Japan, TOMY MX-307) at 25 °C, 120 rpm, and then the supernatant solution was extracted and stored in a −80 °C refrigerator (Sanyo Electric Co., Ltd., Osaka, Japan, SANYO MDF-C8V).

On the day of analysis for each sample, they were taken out of the −80 and put on racks to wait for melting. After the solutions melted, 80 µL of them were put in a new 1.5 mL tube. The centrifugal evaporation machine (Tokyo Rikakikai Co. Ltd. (EYELA), Tokyo, Japan, EYELA CVE-3110) was turned on 30 min before the samples were input there, and, after that, the machine was turned on, and a period of 2 h passed. Proceeding with the use of the centrifugal evaporator machine, the freeze-drier machine (Tokyo Rikakikai Co. Ltd. (EYELA), Tokyo, Japan, EYELA FDM-100) was used at −40 °C for an overnight period.

On the next day, the machine was turned off, and, when the temperature inside the machine reached ambient temperature, the samples were removed from it. 40 µL of MACl (20 mg of Methoxyamine hydrochloride +1 mL of pyridine solution) were put into each sample, and then they were put into a thermomixer (Eppendorf AG, Hamburg, Germany, Eppendorf thermomixer F2.0) for 90 min at 37 °C and 1200 rpm. In sequence, 50 µL of MSTFA (N-Methyl-N-trimethylsilyltrifluoroacetamide) was added to each sample, they were centrifuged in the small centrifuge (Nichiryo Co., Ltd., Tokyo, Japan, NICHIRYO C1008-B), and were put in the thermomixer (Eppendorf thermomixer F2.0) again for 30 min at 37 °C, 1200 rpm.

Proceeding from the last step, 50 µL of the solution was extracted and put into small bottles made for the GCMS procedure. The GCMS (Gas Chromatograph-Mass Spectrometer) machine (Shimadzu Corporation, Kyoto, Japan, SHIMADZU GC-2010) was then prepared according to the experiment and executed to gain results for 50 metabolites, 9 of which were chosen as the most significant ones and discussed below. After finding the concentrations of the metabolites, a Principal Component Analysis (PCA) was performed with the statistic software XLSTAT 2025, utilizing the data of all 50 metabolites tested (pyruvic acid, alanine, oxalic acid, valine, urea, leucine, isoleucine, proline, succinic acid, glycine, fumaric acid, citraconic, serine, threonine, malic acid, threitol, aspartic acid, methionine, hydroxyprolisilane, aminobutyl, cysteine, ketoglutarate, glutamic acid, phenylalanine, asparagine, glutamine, aconitic acid, putrescine, glutamine, fructose, ornithine, citric acid, fructose, histamine, glucose, lysine, tyrosine, sorbitol, galacturonic, inositol, tryptophan, tryptamine, sucrose, 1-aminocyclopropane-1-carboxylic acid (ACC), caffeine, and theanin).

## 5. Conclusions

In conclusion, the study suggests that GB acts as a key osmoprotectant in passion fruit under drought conditions, supporting the plant’s ability to maintain turgor and metabolic function. The seedlings showed a better overall response to glycine betaine application and stress adaptation, while cuttings showed greater chlorophyll stability (SPAD values) and transpiration adjustments, indicating that different propagation methods at different phenological stages impact physiological responses to abiotic stresses in the early phase of development. Moreover, glycine betaine application also raised the concentration of important stress-related substances such as leucine, valine, malic acid, and soluble sugars, which are important metabolites that protect plants against abiotic stress.

Glycine betaine, in this study, has been shown to be a good substance for the mitigation of drought, but some negative impacts were also found. It was found that extensive application for long periods of time may damage the plants and impair their growth, proving the need for further studies on the concentration and application frequency of amino acid for better and safer results.

Overall, the analysis demonstrates that glycine betaine substantially improves drought tolerance by enhancing metabolic flexibility and activating pathways associated with energy production, nitrogen assimilation, and osmoprotection. Nonetheless, the right conditions and application methods of the substance should be studied to minimize any possible damage and increase the positive consequences.

## Figures and Tables

**Table 1:** Effect of GB application on leucine, valine, succinic acid, malic acid, threitol, aminobutyl, citric acid, glucose, and proline concentration values of cuttings and seedlings of passion fruit. “S” represents seedlings, “C” stands for cuttings, “D0” refers to control drought, “D1” indicates mild drought, and “D2” denotes severe drought. “GB0” indicates no application of glycine betaine, whereas “GB1” signifies glycine betaine application.

			Metabolite Concentration (μmol/g)				
			Leucine	Valine	Succinic Acid	Malic Acid	Threitol
Treatments	Cutting	D0GB0	1.04 ± 0.09 b	0.81 ± 0.07 b	0.35 ± 0.01 e	65.51 ± 1.64 f	0.90 ± 0.02 c
		D0GB1	1.15 ± 0.11 a	0.91 ± 0.05 b	1.14 ± 0.11 a	133.70 ± 11.72 c	0.55 ± 0.04 f
		D1GB0	0.08 ± 0.02 e	0.03 ± 0.02 g	0.81 ± 0.08 b	93.57 ± 3.83 e	0.66 ± 0.05 d
		D1GB1	0.67 ± 0.05 c	0.50 ± 0.05 d	0.48 ± 0.04 e	164.09 ± 5 b	1.16 ± 0.06 b
		D2GB0	0.01 ± 0.00 e	0 ± 0 g	0.33 ± 0.05 f	65.02 ± 14.06 f	1.29 ± 0.05 a
		D2GB1	0.34 ± 0.03 d	0.21 ± 0.04 f	0.54 ± 0.05 d	108.27 ± 6.82 d	1.08 ± 0.13 b
	Seedling	D0GB0	0.96 ± 0.04 b	0.30 ± 0.06 e	0.33 ± 0.01 f	113.71 ± 3.9 d	0.45 ± 0.02 f
		D0GB1	1.32 ± 0.02 a	1.17 ± 0.02 a	0.80 ± 0.08 b	172.97 ± 3.83 b	0.61 ± 0.05 e
		D1GB0	1.05 ± 0.08 a	0.63 ± 0.02 c	0.64 ± 0.11c	175.81 ± 7.74 b	0.42 ± 0.06 f
		D1GB1	1.41 ± 0.11 a	0.93 ± 0.03 a	0.87 ± 0.06 a	231.75 ± 2.63 a	0.37 ± 0.05 f
		D2GB0	0.64 ± 0.12 c	0.62 ± 0.05 c	0.58 ± 0.02 c	89.5 ± 7.60 e	0.94 ± 0.09 c
		D2GB1	0.52 ± 0.08 d	0.64 ± 0.08 c	0.99 ± 0.11 a	187.22 ± 6.91 b	1.57 ± 0.10 a
			**Aminobutyl**	**Citric Acid**	**Glucose**	**Proline**	
	Cutting	D0GB0	2.27 ± 0.36 e	11.89 ± 0.83 d	84.01 ± 0.99 c	14.35 ± 1.46 c	
		D0GB1	6.64 ± 0.45 b	58.51 ± 4.27 a	72.24 ± 4.19 d	20.09 ± 1.38 b	
		D1GB0	0.32 ± 0.14 e	21.16 ± 1.59 c	87.53 ± 10.18 b	0 ± 0 e	
		D1GB1	4.94 ± 0.55 c	9.69 ± 0.42 e	99.43 ± 6.93 b	0 ± 0 e	
		D2GB0	0 ± 0 f	0.51 ± 0.06 f	91.06 ± 0.99 b	0 ± 0 e	
		D2GB1	5.19 ± 0.64 c	16.20 ± 1.13 d	65.44 ± 4.85 e	0 ± 0 e	
	Seedling	D0GB0	2.24 ± 0.13 e	27.41 ± 1.37 b	60.67 ± 3.41 f	0 ± 0 e	
		D0GB1	9.41 ± 0.14 a	33.29 ± 1.59 b	145.24 ± 10.18 a	31.42 ± 2 a	
		D1GB0	5.90 ± 0.38 b	49.14 ± 3.37 a	107.00 ± 3.66 b	11.84 ± 2.34 c	
		D1GB1	8.87 ± 0.31 a	32.89 ± 3.46 b	142.00 ± 5.79 a	30.57 ± 2.90 a	
		D2GB0	4.50 ± 0.55 d	11.56 ± 0.45 d	69.25 ± 2.75 d	7.15 ± 0.9 d	
		D2GB1	7.74 ± 0.40 a	20.46 ± 3.81 c	110.42 ± 8.28 b	0 e	

Different letters mean different significant results with a Tukey at 5%. “S” represents seedlings, “C” represents cuttings, “D0” refers to control (no drought), “D1” indicates mild drought, and “D2” denotes severe drought. “GB0” corresponds to no glycine betaine application, whereas “GB1” signifies glycine betaine application.

## Data Availability

The data presented in this study are available on request from the corresponding author.

## Abbreviations

The following abbreviations are used in this manuscript:

GB	Glycine betaine
LWP	Leaf water potential
PCA	Principal component analysis
bZIPs	Basic leucine zipper transcription factors
BCAA	Branched-chain amino acids
GABA	Gamma-aminobutyric acid
MACl	Methoxyamine hydrochloride
MSTFA	N-Methyl-N-trimethylsilyltrifluoroacetamide
GCMS	Gas chromatograph–mass spectrometer
ACC	1-aminocyclopropane-1-carboxylic acid
C	Cuttings
S	Seedlings
D0	Control drought (no water deficit)
D1	Mild drought
D2	Severe drought
GB0	No application of glycine betaine
GB1	Application of 100 mM glycine betaine
